# Regions of the amino terminus of the P2X_1_ receptor required for modification by phorbol ester and mGluR1α receptors

**DOI:** 10.1111/j.1471-4159.2008.05761.x

**Published:** 2009-01

**Authors:** Hairuo Wen, Richard J Evans

**Affiliations:** Department of Cell Physiology & Pharmacology, University of LeicesterLeicester, UK

**Keywords:** ATP, mutagenesis, P2X receptors, protein kinase C, regulation

## Abstract

The potentiation of P2X_1_ receptor currents by phorbol ester (PMA) treatment and stimulation of mGluR1α receptors was sensitive to inhibition of novel forms of protein kinase C. Potentiation was also reduced by co-expression of an amino terminal P2X_1_ receptor minigene. Cysteine point mutants of residues Tyr^16^-Gly^30^ were expressed in *Xenopus oocytes.* Peak current amplitudes to ATP for Y16C, T18C and R20C mutants were reduced, however this did not result from a decrease in surface expression of the channels. The majority of the mutants showed changes in the time-course of desensitization of ATP evoked currents indicating the important role of this region in regulation of channel properties. PMA and mGluR1α potentiation was abolished for the mutants Y16C, T18C, R20C, K27C and G30C. Minigenes incorporating either Y16C, K27C, V29C or G30C still inhibited PMA responses. However D17C, T18C or R20C mutant minigenes were no longer effective suggesting that these residues are important for interaction with regulatory factors. These results demonstrate that the conserved YXTXK/R sequence and a region with a conserved glycine residue close to the first transmembrane segment contribute to PMA and GPCR regulation of P2X_1_ receptors.

Genes encoding seven mammalian P2X receptor subtypes (P2X_1-7_) have been identified and they assemble to form homo- and hetero-trimeric ATP-gated channels with a range of phenotypes (North 2002). P2X_1_ receptors are expressed throughout the body and contribute to a range of physiological processes, e.g. regulation of smooth muscle tone (Valera *et al.* 1994; Scase *et al.* 1998; Sage *et al.* 2000) and thrombosis (Hechler *et al.* 2003). In the nervous system P2X_1_ receptors form heteromeric assemblies and are involved in the pre-synaptic regulation of transmitter release in the auditory brainstem (Watano *et al.* 2004) and a P2X_1/5_ heteromeric receptor has recently been described in astrocytes (Lalo *et al.* 2008).

P2X receptors constitute a distinct family of ligand gated ion channels with intracellular amino and carboxy termini, two transmembrane segments and a large extracellular loop involved in drug action (Roberts *et al.* 2006). The intracellular regions of the channels have been shown to be involved in channel regulation (Boue-Grabot *et al.*2000; Jiang *et al.*2001; Eickhorst *et al.*2002; Ennion and Evans 2002; Chaumont *et al.* 2004; Vial *et al.* 2004). The amino termini have a relatively standard length of about 30 amino acids (North 2002). This contains a protein kinase C consensus sequence TXK/R preceded by a conserved tyrosine giving rise to a YXTXK/R motif (Tyr^16^-Lys^20^ in the P2X_1_ receptor) that is conserved in all mammalian and Dictyostelim receptors (Boue-Grabot *et al.* 2000; Fountain *et al.* 2007). Mutations of the central threonine led to a speeding of channel desensitization and reduction in peak current amplitude (Boue-Grabot *et al.*2000; Ennion and Evans 2002) and this residue may be constitutively phosphorylated (Liu *et al.* 2003). Furthermore, for the P2X_2_ receptor when the C-terminal was truncated, the P2X_2_ receptor showed faster desensitization, but the normal wild type time-course was recovered by phorbol ester which stimulates PKC (Boue-Grabot *et al.* 2000). Therefore, the N-terminus may be involved in intracellular regulatory mechanisms.

P2X receptors can be regulated by G-protein coupled receptors (GPCRs) (Ralevic and Burnstock 1998; Paukert *et al.* 2001; Kunapuli *et al.* 2003; Vial *et al.* 2004). For example P2X_1_ receptor currents can be potentiated by mGluR1α, P2Y_1_, P2Y_2_ and 5-hydroxytryptamine (5-HT)_2A_ receptors as well as by phorbol ester (phorbol-12-myristate-13-acetate, PMA) treatment and these effects were abolished by the broad spectrum kinase inhibitor staurosporine (Vial *et al.* 2004; Ase *et al.* 2005). This was independent of phosphorylation of the consensus PKC site, as potentiation was still seen when the conserved threonine residue was mutated, and it has been suggested that the modulatory effects may result from action on an interacting protein (Vial *et al.* 2004). However amino acids in the P2X_1_ receptor that contribute to the regulation were not determined.

In this study, we have investigated (i) the role of novel, calcium insensitive, protein kinase C isoforms in the control of the P2X_1_ receptor, (ii) the contribution of the N-terminus of the P2X_1_ receptor in regulation using over-expression of a minigene and (iii) used cysteine scanning of the 15 residues before the first transmembrane segment to identify for the first time residues involved in regulation of P2X_1_ receptors by GPCRs and phorbol ester.

## Methods

### Minigene construction

The amino terminal sequence (Met^1^-Gly^30^) of the human P2X_1_ receptor was amplified from the pcDNA 3.0 vector containing the human P2X_1_ receptor cDNA by Polymerase Chain Reaction (PCR) (Techne Genius thermocycler, BioTAQTM DNA polymerase, Bioline, UK). Start and stop codons at the ends of the minigene as well as restriction sites, EcoRI and HindIII, were introduced using the primers. The minigene sequence was ligated into the plasmid pcDNA3.0 using these two restriction sites at 14°C overnight (T4 DNA ligase, New England Biolabs® Inc., Hertfordshire, UK).

### Site-directed mutagenesis

Point mutations were introduced into the human P2X_1_ plasmid or the minigene construct using the QuikChangeTM mutagenesis kit (Stratagene, Amsterdam, Netherlands) according to the manufacturer’s instructions as described previously (Ennion *et al.* 2000) and confirmed by DNA sequencing (Automated ABI Sequencing Service, Leicester University, Leicester, UK).

### Expression in xenopus laevis oocytes

The human mGluR1α receptor was a gift from Professor S. R. Nahorski (University of Leicester, Leicester, UK). pcDNA3.1 vectors (Invitrogen, Paisley, UK) containing either P2X_1_ mutant, wild-type P2X_1_, mGluR1α receptors or the N-termini minigene were linearized. Sense-strand cRNAs were generated from these linearized plasmids with the T7 mMessage mMachineTM kit [Ambion (Europe), Huntingdon, Cambs., UK].

*Xenopus laevis oocytes,* stage V, were prepared by enzymatic treatment followed by manual defoliculation as described previously (Ennion *et al.* 2000). 50 nL of mRNA (1 μg/μL) was injected into isolated *Xenopus oocytes* using an Inject+Matic microinjector (J.Alejandro Gaby, Geneva, Switzerland). For co-injections with N-termini minigenes the RNA was mixed to give 5 ng wild type (WT) P2X_1_+10 ng mGluR1α + 35 ng N-termini minigene (or appropriate volume of water was added in the absence of minigene) and injected in a 50 nL volume. Cells were maintained at 18°C in ND96 buffer (concentrations in mM; 96 NaCl, 2 KCl, 1.8 CaCl_2_, 1 MgCl_2_, 5 sodium pyruvate and 5 HEPES, pH 7.5) with 50 μg/mL gentamicin and were used for recording after 2–6 days.

### Electrophysiological recordings

Two-electrode voltage clamp was used on cRNA-injected *oocytes* to record currents to applied ATP (Mg salt; Sigma, Poole, UK) as described previously (Ennion *et al.* 2000). ATP was applied with a fast-flow U-tube perfusion system, applications of ATP were separated by 5 min in order to allow recovery from receptor desensitization.

For *oocytes* pre-treated with PMA, 100 nM PMA was made in ND96 solution and the *oocytes* were pre-incubated in the PMA solution for 10 min at 21°C immediately before recording. Comparisons were made between groups of control untreated *oocytes* and those exposed to PMA. The protein kinase inhibitors Calphostin C(1 μM) (Sigma C6303), K252a (100 nM) (Sigma 05288), Gö6983(200 nM) (Sigma G1918) or Gö6976 (200 nM) (Sigma G1171) were applied to the *oocytes* for 1 h at 21°C before recording. The inhibitors, when applied alone had no effect on the peak current amplitude or the time-course of P2X_1_ receptor currents (data not shown). When looking at the potentiation of the P2X_1_ receptor, glutamate (100 μM) with or without the protein kinase inhibitors was bath-perfused for 5 min between the stimulations of the P2X_1_ receptor by ATP via the U-tube as described previously. The glutamate was applied to the oocytes when stable responses were observed (Vial *et al.* 2004).

### Western-blotting

The expression levels and molecular weight of the P2X receptor proteins were estimated by western blotting, and both the level of expressed total and cell surface P2X_1_ receptors were studied as described previously (Ennion *et al.* 2000).

### Data analysis

All data are shown as mean ± SEM Significant differences between the means of all groups compared to WT were calculated by one-way anova, followed by Dunnett’s test for comparisons of individual mutants against control using the GraphPad Prism 5 for Windows (GraphPad Software, San Diego, CA, USA). Student’s *t* tests were also used where appropriate and considered to be significant when *p*< 0.05. *n* corresponds to the number of *oocytes* tested for electrophysiological data.

## Results

### Novel protein kinase C isoforms contribute to regulation of the P2X_1_ receptor by PMA and GPCRs

**Fig. 1:**
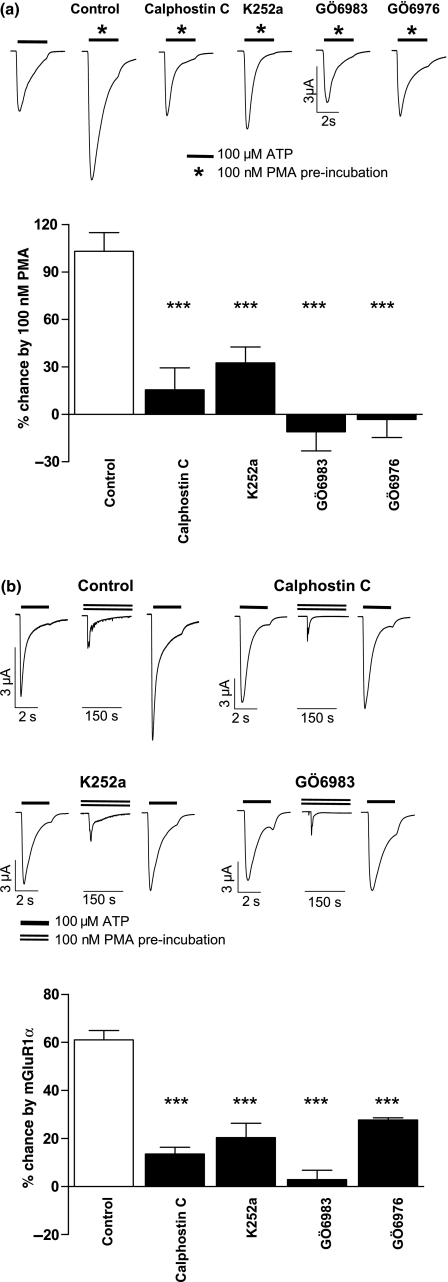
Potentiation of P2X_1_ receptor currents by PMA and mGluR1α receptor stimulation is sensitive to inhibitors of novel isoforms of protein kinase C. (a) Representative traces of currents evoked by ATP (100 μM) under control conditions (left) and following treatment with PMA (100 nM) or PMA following incubation with the PKC inhibitors (1 h pre-incubation before 10 min of PMA) calphostin C (1 μM), K252a (100 nM), Gö6983 (200 nM) or Gö6976 (200 nM). The lower panel shows a summary of the effects of the inhibitors on PMA potentiation, *n*=5–19. (b) Sample traces of the effects of PKC inhibitors and mGluR1α mediated potentiation of P2X_1_ receptor currents. Application of glutamate (100 μM) evoked a transient inward calcium activated chloride current and potentiated the subsequent ATP current. Potentiation was reduced following pre-treatment of the oocytes with the PKC inhibitors. The lower panel shows a summary of the effects, of the inhibitors on glutamate potentiation, *n*=3–13. ***p< 0.001.

For *oocytes* co-expressing P2X_1_ and mGluR1α receptors ATP (100 μM, a maximal concentration) evoked transient inward currents that desensitised during the application of ATP. P2X_1_ receptor currents were potentiated by 103.1 ± 11.8% (*n*=19) following 10 min pre-treatment with PMA (100 nM) (Fig. 1). Similarly, the activation of mGluR1α receptors by 100 μM glutamate for 5 min evoked a transient inward calcium activated chloride current and potentiated the subsequent responses of WT P2X_1_ receptors to ATP (100 μM) by 61.0 ± 3.9% (*n*=13) (Fig. 1). This is consistent with our previous studies on the P2X_1_ receptor (Vial *et al.* 2004). We have previously shown that these effects could be reduced by treatment with the broad spectrum kinase inhibitor staurosporine but were unlikely to involve classical isoforms of protein kinase C as potentiation was not calcium sensitive (Vial *et al.* 2004). To determine whether novel, calcium insensitive, protein kinase C isoforms (PKCδ,ε,η,θ, or μ) are involved in the regulation we have used a range of inhibitors (Fig. 1) [none of these had an effect on peak P2X_1_ receptor current amplitude or time course (data not shown)]. Calphostin C(1 μM) acts by competing with the diacyl glycerol and phorbol ester binding site of protein kinase C isoforms and reduced potentiation by PMA and mGluR1α stimulation to 15.5 ± 13.9% (*n*=11) and 13.5 ± 2.8% (*n*=5). Potentiation was also reduced by the kinase inhibitor K252a (100 nM) (32.6 ± 10.0% and 20.3 ± 5.9%, *n*=19, 5 respectively). Gö6983 is a kinase inhibitor at novel PKCs δ,ε,η,θ, but is ineffective at the novel isoform PKCμ (also called protein kinase D, PKD)(Martiny-Baron *et al.* 1993). Potentiation was reduced to −6.9 ± 12.4% (*n*=13) and −2.8 ± 3.9% (*n*=7) by Gö6983 (200 nM) for PMA and mGlur1α respectively. Gö6976 (200 nM) is an effective inhibitor of the novel calcium insensitive protein kinase C isoform PKCμ but is ineffective at the other calcium insensitive novel PKC isoforms (Gschwendt *et al.* 1996) and reduced PMA and mGluR1α mediated potentiation to −3.2 ± 11.4% (*n*=8) and 27.6 ± 0.9% (*n*=3). These findings are similar to those reported for 5-HT dependent regulation of the receptor (Ase *et al.* 2005) and suggest that novel protein kinase C isoforms mediate P2X_1_ receptor regulation by both PMA and GPCRs. However our previous studies showed that P2X_1_ receptor current potentiation was unaffected by mutation to remove the consensus PKC site, and a change in the phosphorylation status of the receptor was not detected (Vial *et al.* 2004). These results suggest that the potentiation is mediated by phosphorylation of an interacting regulatory protein (Vial *et al.* 2004).

### The amino terminus of the P2X_1_ receptor is involved in GPCR and phorbol ester regulation

**Fig. 2:**
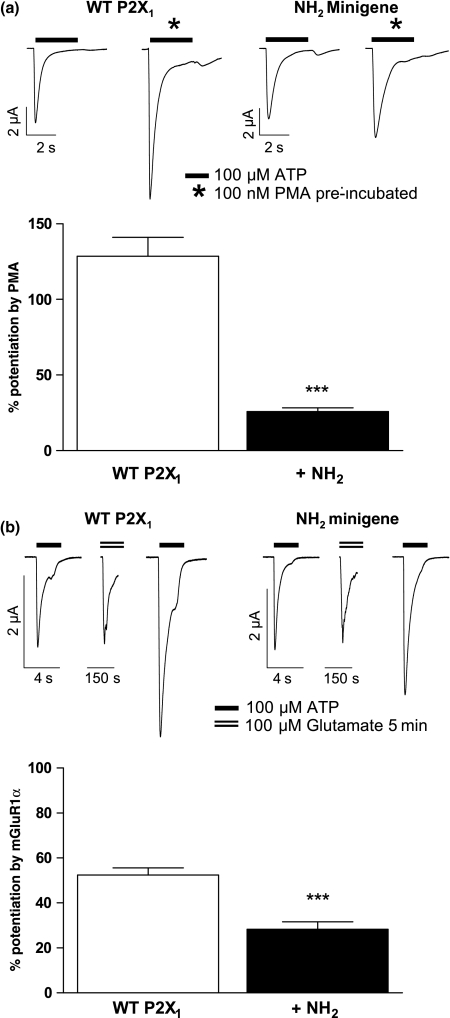
The N-termini P2X_1_ receptor minigene blocks the potentiating effects of PMA and mGluR1α receptor stimulation on P2X_1_ receptor currents. A minigene encoding the N-terminal sequence of the P2X_1_ receptor was co-expressed with wild type P2X_1_ and mGluR1α receptors in the *Xenopus oocytes*. (a) Upper left panels show representative currents evoked by a maximal concentration of ATP (100 μM, indicated by bar) at control *oocytes* (WT P2X_1_) and those following 10 min incubation with PMA (100 nM). Right upper panels show the effects of co-expression of the amino terminal minigene (NH_2_ minigene) on the effects of PMA. The bar chart shows summary data, *n*=6–7. (b) Upper panels show sample traces for a given *oocyte* co-expressing P2X_1_ and mGluR1α receptors (left) or P2X_1_ receptors, mGluR1α receptors and the P2X_1_ receptor amino terminal minigene (right traces). Responses to a maximal concentration of ATP (100 μM, indicated by bar) are shown before and after the application of glutamate (100 μM). Glutamate evoked an inward calcium activated chloride current and potentiated subsequent ATP evoked responses. This potentiation was reduced by co-expression of the P2X_1_ receptor N-terminal minigene. The bar chart shows a summary of the data, *n*=6–7. ***p< 0.001.

To determine whether the intracellular amino terminus of the P2X_1_ receptor contributes to receptor modulation we co-expressed P2X_1_ receptors and mGluR1α receptors with a minigene encoding the amino terminus of the P2X_1_ receptor. P2X_1_ receptor amino terminal minigene expression had no effect on the P2X_1_ receptor currents (peak current amplitudes to 100 μM ATP of −6875 ± 298 nA and −6302 ± 304 nA, for control and with the minigene respectively *n*=6), or the amplitude of glutamate evoked chloride currents (−4996 ± 807 nA and −4155 ± 1233 nA respectively) demonstrating that the minigene does not regulate P2X_1_ or mGluR1α receptor expression or activation (Fig. 2). However co-expression of the amino terminal minigene reduced potentiation by PMA from 128.5 ± 12.6% to 25.8 ± 2.6% (*p*< 0001, *n*=6, 7) and by mGluR1α receptor stimulation from 52.5 ± 3.1% to 28.3 ± 3.3% (*p*< 0.001, *n*=6, 7) (Fig. 2). This suggests that the amino terminus is important in regulation and that the minigene may work by sequestering regulatory factor(s) associated with the receptor.

### Effects of point cysteine substitutions on basic P2X_1_ receptor properties

**Fig. 3:**
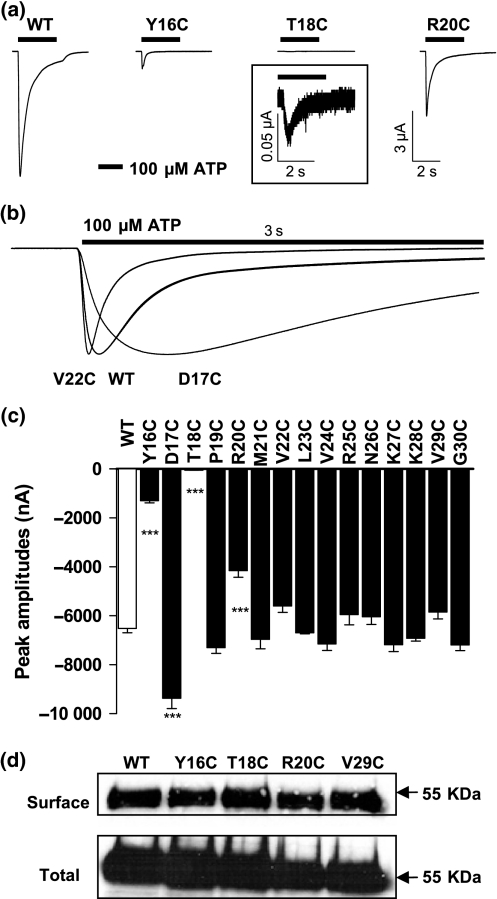
The basic properties of cysteine points mutants of the P2X_1_ receptor N-terminal. (a) ATP (100 μM) evoked rapidly desensitising responses at WT P2X_1_ receptors. Desensitising responses were also recorded for the mutants Y16C, T18C and R20C however these were of reduced amplitude. For T18C an insert is provided showing at an increased scale the time-course of the ATP evoked response. (b) Peak current normalised traces showing the more rapid (V22C) and slower (D17C) rates of channel desensitisation of ATP evoked responses compared to WT. (c) Peak current amplitudes of WT and P2X_1_ receptor mutants to ATP (100 μM). ***p< 0.001. (d) Surface and total expression levels of WT and mutant P2X receptors with reduced peak current amplitudes.

We used cysteine substitution mutagenesis to investigate the contribution of the 15 amino acids before the first transmembrane segment to channel properties. This region includes the conserved YXTXK/R motif and SCAM (substituted cysteine accessibility method) analysis of P2X_2_ receptors suggested that this part of the amino terminal may play a role in channel function (Jiang *et al.* 2001). ATP (100 μM) evoked fast desensitizing inward currents from all of the mutants. There was no effect on the peak current amplitude for 12 of the mutants (Fig. 3, Table 1) however responses were reduced for mutants Y16C, T18C, and R20C (Fig. 3, Table 1). Western blotting showed there was no obvious difference in either total or surface expression levels for these mutants compared to WT (Fig. 3) demonstrating that the reduction in current amplitude does not result from deficiencies in receptor trafficking.

**Table 1:** Summary of basic properties of P2X_1_ receptor cysteine mutants

P2X_1_ receptor	Peak response at 100 μM ATP (nA)	100–50% decay time (ms)
WT	−6513 ± 179	258 ± 7
Y16C	−1297 ± 93***	232 ± 13
D17C	−9361 ± 429***	1501 ± 12***
T18C	−52 ± 4***	146 ± 10***
P19C	−7302 ± 235	1070 ± 20***
R20C	−4153 ± 273***	244 ± 4
M21C	−6962 ± 389	409 ± 13***
V22C	−5605 ± 255	87 ± 6***
L23C	−6698 ± 41	528 ± 20***
V24C	−7147 ± 265	284 ± 11
R25C	−5950 ± 419	165 ± 9***
N26C	−6039 ± 319	241 ± 12
K27C	−7182 ± 279	339 ± 26*
K28C	−6921 ± 113	666 ± 21***
V29C	−5849 ± 277	169 ± 4***
G30C	−7190 ± 232	242 ± 10

The peak current amplitude and the decay time from 100% to 50% of the peak current are summarized for the WT P2X_1_ receptor and the cysteine mutants (*n*=4–21).

* *p*< 0.05

*** *p*< 0.001.

There were no major changes in ATP potency at the mutant receptors; 1 μM ATP, an ∼ EC_50_ concentration at the WT receptor, evoked between 30% and 70% of the maximum current amplitudes for each of the mutants. The time-course of desensitisation (time for the peak currents to 100 μM ATP to decay to 50%) was unaffected for the mutants Y16C, R20C, V24C, N26C and G30C. Gly^30^ is conserved throughout the mammalian and *Dictyostelium* families (Fountain *et al.* 2007). However, it is interesting that mutation to cysteine had no effect on the current amplitude of time-course of P2X_1_ (this study) or P2X_2_ (Jiang *et al.* 2001) receptor currents. This shows that the flexibility associated with the glycine residue is not essential for normal channel function. For the remainder of the mutants, there were significant changes in time-course. Slowed desensitisation was recorded for D17C, P19C, L23C and K28C; with D17C responses slowed almost 6 fold (Fig. 3, Table 1). Faster desensitisation was seen for the remainder of the mutants with the greatest change seen for V22C with an ∼ 3 fold speeding (greater than for T18C) in the decay of current evoked during the continued presence of ATP but no effect on peak current amplitude (see Fig. 3b, Table 1). Studies on the P2X_2_ receptor showed for the majority of analogous mutants (13/15) there was no effect on the time-course of the response (Jiang *et al.* 2001). This may reflect that P2X_2_ receptor currents are relatively non-desensitising whereas for the P2X_1_ receptor there is a rapid transition once the channel is open to the desensitised state, and this is more sensitive to disruption of the amino terminus.

### Cysteine substitution can block PMA potentiation

**Fig. 4:**
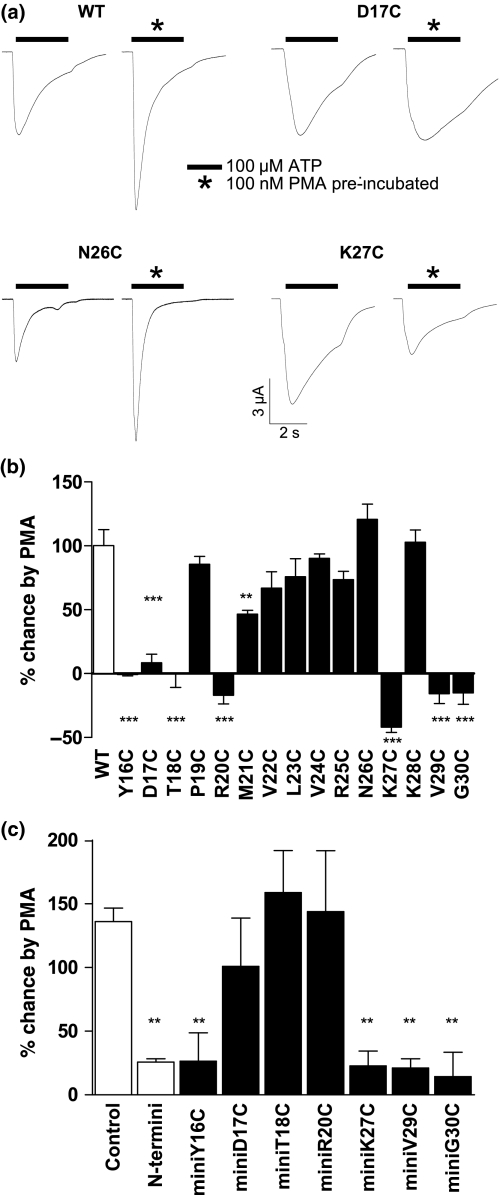
PMA potentiation can be abolished by cysteine substitution of amino terminal residues. (a) Sample traces of ATP evoked currents (100 μM application indicated by bar) from *oocytes* under control conditions and following PMA (100 nM) treatment for WT as well as the mutants D17C, N26C and K27C. (b) Summary of the percentage changes of peak amplitudes by PMA treatment for N-termini cysteine mutants. Cysteine mutants around the conserved PKC consensus site and next to the first transmembrane segment were no longer potentiated by PMA. (c) Effects of mutations of the minigene on PMA potentiation. **p< 0.01, ***p< 0.001.

WT P2X_1_ receptors were potentiated by 116.5 ± 14.5% (*n*=11) following 10 min application of PMA. A similar level of potentiation was seen for the mutants P19C, V22C, L23C, V24C, V25C, N26C and K28C. For M21C, potentiation was reduced to about 50%, no potentiation was seen for the mutants Y16C, D17C, T18C, R20C, V29C and G30C (Fig. 4), and the responses were inhibited for mutant K27C. These results suggest a cluster of residues comprising the conserved YXTXK/R motif and those close to the first transmembrane segment of the P2X_1_ receptor are involved in PMA regulation.

### Mutant minigenes identify residues important in interaction with regulatory factors

Cysteine mutants that reduced PMA potentiation could be important for mediating the interaction with a regulatory factor and/or important in conformational changes induced by the regulatory factor. To address these roles we introduced the individual cysteint mutants that reduced PMA potentiation into the N terminal minigene. It was predicted that mutation of residues in the minigene important for interaction with regulatory factors/proteins would remove the inhibitory/sequestering effect of the minigene. Minigenes expressing the mutants D17C, T18C and R20C no longer inhibited the PMA induced potentiation (Fig. 4) consistent with a role of these residues in mediating interactions with regulatory factors. It is unlikely that these effects result from the disruption of the protein kinase C consensus in the minigene as PMA is still effective at potentiating P2X_1_ receptors where the consensus for phosphorylation has been mutated (Vial *et al.* 2004, and this study). In contrast Y16C, K27C, V29C and G30C mutant minigenes still inhibited PMA potentiation and suggested that these residues may be important in conformational changes on the P2X_1_ receptor associated with PMA potentiation.

### Differential sensitivity of cysteine mutants to mGluR1α receptor and PMA

**Fig. 5:**
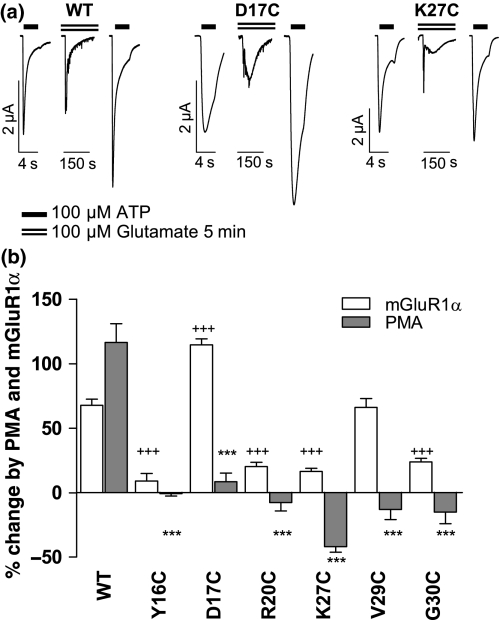
The effects of mGluR1α receptor activation on P2X_1_ receptor mutants. (a) Sample traces for a given *oocyte* co-expressing either WT P2X_1_, D17C or K27C mutant P2X_1_ receptor with mGluR1α receptors. Responses to a maximal concentration of ATP (100 μM, indicated by bar) are shown before and after the application of glutamate (dotted line). Glutamate (100 μM) evoked an inward calcium activated chloride current and potentiated subsequent ATP evoked responses for WT and D17C mutants but not for the K27C mutant P2X_1_ receptor. (b) The effects of mGluR1α receptor (100 μM glutamate) and PMA (100 nM) on WT and the cysteine mutants are shown (+++*p*< 0.001 comparing mGluR1 receptor regulation of mutants to WT, ***p< 0.001 for mutants treated with PMA compared to the WT effect). For most of the mutants unable to exhibit the PMA potentiation, no potentiation was seen following the activation of mGluR1α receptor. However, the mGluR1α receptor stimulated potentiation was still robust for the D17C and V29C mutants.

For the mutants where PMA no longer had an effect or reduced the response, we also tested to see whether mGluR1α receptor dependent regulation was also attenuated. ATP (100 μM) was applied repeatedly at 5 min intervals to obtain reproducible responses. Glutamate potentiated WT P2X_1_ receptor currents by 67.8 ± 4.8% (*n*=8). For most (4/6) of the mutants that showed no potentiation with PMA treatment the effects of mGluR1α receptor stimulation were also reduced significantly (Fig. 5). However, the mGluR1α receptor regulation was not affected by the mutations D17C and V29C (that abolished PMA potentiation) where the application of glutamate gave potent potentiations of 114.5 ± 4.7% (*n*=4) and 66.2 ± 6.9% (*n*=5) respectively. These results demonstrate that PMA and mGluR1α stimulation have some differences in their regulatory mechanisms.

### Substitution dependence on residues compromising the consensus PKC site

**Fig. 6:**
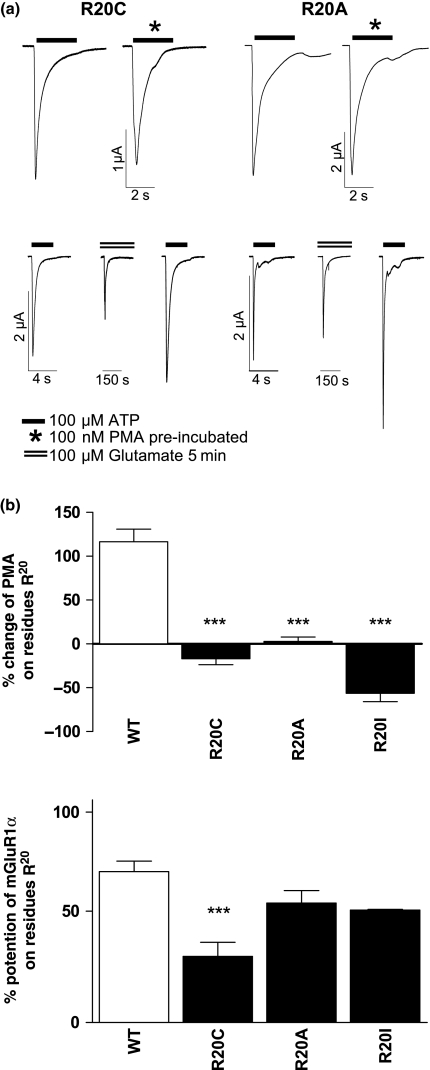
The effects of PMA and mGluR1α receptor to R20 substitutions. (a) Example traces of R20C and R20A mutants in response to PMA (100 nM) or mGluR1α receptor stimulation (100 μM glutamate) are shown. Peak amplitudes from control and PMA treated oocytes are shown. In the lower panels ATP evoked currents before and after mGluR1α receptor activation from either R20C (left) or R20A (right) are shown. (b) Summary of effects of amino acid substitution at position R20 at the P2X_1_ receptor by cysteine, alanine or isoleucine on potentiation by PMA or glutamate. ***p< 0.001.

The consensus PKC motif (TXR/K) is conserved on the N-terminus of P2X receptors. When this consensus was disrupted (R20A) comparable potentiation by GPCRs to the WT P2X_1_ receptor was observed (Vial *et al.* 2004). As the R20C mutant exhibited a dramatic reduction to the effect of mGluR1α receptor stimulation we have determined the effect of different substitutions at residue Arg^20^. Following co-expression with the mGluR1α receptor similar levels of potentiation as for WT receptors were seen for R20A and R20I mutants following application of glutamate (100 μM) (Fig. 6). However in all cases (R20C, R20A and R20I), the effects of PMA were abolished (Fig. 6). These results further highlight differences between PMA and mGluR1α mediated regulation. A dependence of the nature of the amino acid substituted was also shown at position Thr^18^. The mutant T18C blocked the PMA (−0.2 ± 10.5%, *n*=4) regulation of the P2X_1_ receptor, while the T18A mutation had no effect on PMA potentiation (194.7 ± 18.8%, *n*=7). These results demonstrate the effects of mutations that remove the consensus PKC motif are dependent on the amino acid substitution and suggest it is the local amino acid environment, and not the ability to be phosphorylated that regulates channel function in this region.

### PMA treatment had no effect on channel permeability

The permeability of some P2X receptors changes with time (Khakh *et al.* 1999; Virginio *et al.* 1999) and can lead to an increase in currents for the P2X_4_ receptor (Khakh *et al.* 1999). TRPV1 receptors show a similar change in permeability on prolonged agonist application and this is potentiated by activation of PKC (Chung *et al.* 2008). We tested whether the potentiation in current at the P2X_1_ receptor following PMA treatment resulted from an increase in permeability. Under control conditions when dimethylamine was the only external cation P2X_1_ receptor currents reversed at −10.2 ± 0.9 mV (*n*=5) (consistent with previous studies on the receptor, Evans *et al.* 1996). The reversal potential for dimethylamine was unaffected (−11.1 ± 1.9 mV, *n*=8) following PMA treatment demonstrating that the potentiation of P2X_1_ receptor currents does not result from an increase in channel permeability.

## Discussion

P2X_1_ receptor mediated responses can be potentiated by phorbol ester and Gα_q_ coupled GPCR stimulation and this may allow for regulation of P2X receptor signalling (Vial *et al.* 2004). The > 80% reduction by calphostin C of P2X_1_ receptor potentiation by PMA or mGluR1α stimulation supports the role of protein kinase C in receptor regulation. Our previous study showed that potentiation was not dependent on a rise in intracellular calcium (Vial *et al.* 2004) and suggests that novel, calcium insensitive, and not classical calcium sensitive PKC isoforms are involved. Amongst the novel PKC isoforms Gö6976 is selective for PKCμ (PKD) and abolished potentiation by PMA and reduced by ∼ 60% mGluR1α modulation. This indicates a role of PKCμ (PKD) in the regulation of P2X_1_ receptors, as reported previously for 5-HT regulation (Ase *et al.* 2005). The difference in effectiveness of the PKCμ inhibitor between PMA and mGluR1α stimulation suggests that there are differences in the regulatory pathways used. This is consistent with mutagenesis studies that showed D17C and V29C mutants abolished PMA potentiation but had no effect on the mGluR1α stimulation. In addition the minigene had a greater inhibitory effect on PMA stimulation than on that evoked by mGluR1α stimulation even though the extent of PMA potentiation at ∼ 120% was significantly higher that the ∼ 55% potentiation evoked by mGluR1a stimulation. This suggests that mGluR1α stimulation recruits additional regulatory pathways compared to PMA. The reduction in potentiation by Gö6983 (effective at the concentration used at novel PKC isoforms with the exception of PKCμ; IC_50_ 100 times higher than that used in the current study) suggests that one or more of the isoforms PKC δ,ε,η, θ are also involved. mGluR1α potentiation was still observed when the proposed ‘phosphorylatable’ threonine was mutated or the consensus PKC sequence was disrupted by Arg^20^ mutation (this study and Vial *et al.* 2004). In addition we could not detect any changes in P2X_1_ receptor phosphorylation on PMA stimulation and studies on the P2X_3_ receptor also failed to detect receptor phosphorylation associated with PKC regulation (Franklin *et al.* 2007). These studies rule out a role of phosphorylation at the consensus PKC site as a mechanism of action and raise the question where the novel PKC isoforms act? Interacting proteins that regulate the function of P2X_7_ and P2X_2_ receptors have been described (Adinolfi *et al.* 2003; Masin *et al.* 2006) and we suggest that modulation of the P2X_1_ receptor occurs through the phosphorylation by a novel PKC of a regulatory protein that interacts with the P2X_1_ receptor.

Minigenes have been used widely to study regulation (Del Gatto *et al.* 1996; Wu *et al.* 2004) including work on P2X receptors (Boue-Grabot *et al.* 2000). The over-expression of a minigene encoding the amino terminus of the P2X_1_ receptor reduced PMA and mGluR1α receptor potentiation of P2X_1_ receptor currents and demonstrated that the amino terminus plays an important role in receptor regulation possibly through providing a docking site for a regulatory protein and therefore over-expression of the minigene led to sequestering of this regulatory protein. The minigene, like the range of PKC inhibitors used, had no effect on the P2X_1_ receptor currents under resting conditions suggesting that the receptor is not basally regulated, and that PMA and GPCR stimulation provide an auxiliary mechanism to modify channel properties. The greater reduction on the PMA effect compared to GPCR stimulation by the amino terminal minigene suggests that GPCR stimulation could have additional regulatory effects (and is consistent with mutations that abolish PMA effects with little action on mGluR1α stimulation). These results also suggest that the C terminus may also play a significant role in the regulation of the P2X_1_ receptor (this is supported by studies with a C terminal minigene H. W. and R. J. E., unpublished observations). In addition the abolition of PMA potentiation by the mutations D17C, R20A and V29C but no effect on mGlur1α stimulation indicates further that there are subtle differences in the regulation following PMA and mGlur1α stimulation, however the molecular basis of this remains to be elucidated.

The cysteine mutagenesis of the P2X_1_ receptor amino terminal identified residues that were important for PMA regulation. The introduction of these mutants into the minigene in a second round of mutagenesis identified substitutions that abolished the inhibitory effect of the minigene (D17C, T18C and R20C) and those that had no effect on the inhibitory actions of the minigene (Y16C, K27C, V29C and G30C). The abolition of the inhibitory effect of the minigene when Asp^17^, Thr^18^ or Arg^20^ were mutated demonstrates that these residues are likely to play an important role in the sequestering action of the minigene in competing with the P2X_1_ receptor for the regulatory factor/protein and identifies for the first time residues that involved in direct association with the regulatory factor/protein. The results with the minigene also show that resides Tyr^16^, Lys^27^, Val^29^ and Gly^30^ are not important in the interaction directly with the regulatory factor but mediate the changes in the P2X_1_ receptor that lead to potentiation of the response. One possibility is that these residues could contribute to the gating of the P2X_1_ receptor or regulation of channel conductance. Responses at the Y16C, P19C and G30C mutants were reduced by cysteine reactive MTS reagents for the P2X_1_ (see Fig. S1; Appendix S1) and this is consistent with the findings for the P19C and G30C mutants at the P2X_2_ receptor (Y16C mutant was non-functional see above) (Jiang *et al.* 2001). In previous studies we have shown that MTS reagents that result in a change in ATP potency at P2X_1_ receptors modify the time-course of the currents (Roberts and Evans 2007). The reduction in amplitude by MTSEA of Y16C, P19C and G30C mutant P2X_1_ receptors with no effect on the time-course of the response suggests that these residues are involved in ionic permeation through the channel. Taken together these results give rise to a mechanism whereby following PMA stimulation Asp^17^, Thr^18^ and Arg^20^ are involved in interaction with a regulatory factor that is phosphorylated, and this subsequently results in a change in channel properties involving residues Tyr^16^, Lys^27^, Val^29^ and Gly^30^.

In summary this study has identified two amino terminal regions, YXTXK/R and Lys^27^-Gly^30^ that are involved in regulation by PMA and ionic permeation. This raises the possibility that there may be some interaction between these two regions to regulate channel properties. Structural models of the intracellular regions however will be required for fuller interpretation of these results.

## Abbreviations used

5-HT: 5-hydroxytryptamine
GPCR: G-protein coupled receptor
PKC: protein kinase C
PKD: protein kinase D
PMA: phorbol-12-myristate-13-acetate
WT: wild type

## Supporting Information

**Fig. S1** The effects of MTSEA on N-termini cysteine mutants.

**Appendix S1** Characterization of the effects of MTSEA; The effects of MTS compounds to cysteine mutants.
