# Supplementary figures and images for: Regions of the amino terminus of the P2X1 receptor required for modification by phorbol ester and mGluR1α receptors
Source: J Neurochem. 2009 Jan;108(2):331–40. doi: 10.1111/j.1471-4159.2008.05761.x (PMC2704932; doi:10.1111/j.1471-4159.2008.05761.x)

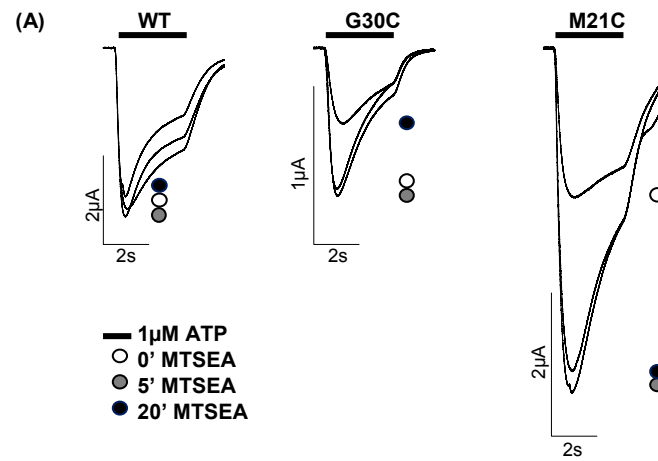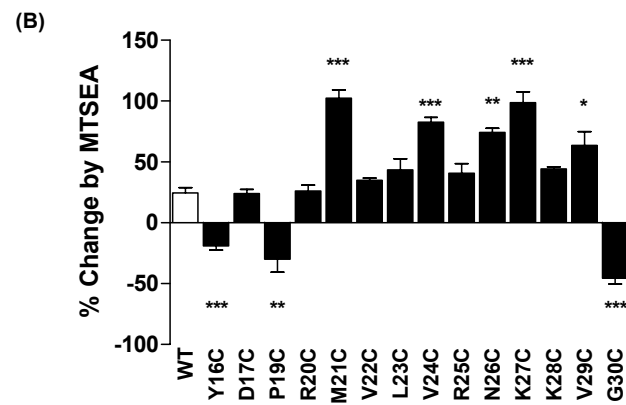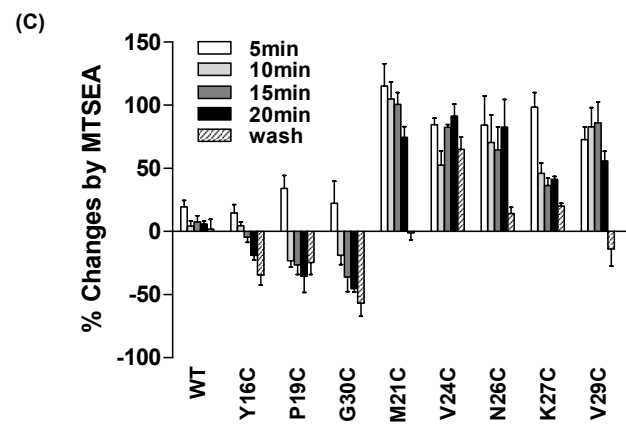

Supplement: Supplementary file 1 [file jnc0108-0331-SD1.pdf]
