# Supplementary material for: Regions of the amino terminus of the P2X1 receptor required for modification by phorbol ester and mGluR1α receptors
Source: J Neurochem. 2009 Jan;108(2):331–40. doi: 10.1111/j.1471-4159.2008.05761.x (PMC2704932; doi:10.1111/j.1471-4159.2008.05761.x)
Supplement: Supplementary file 2 [file jnc0108-0331-SD2.doc]

**Supplementary data**

**Characterization of the effects of MTSEA**

(2-aminoethyl) methanethiosulfonate hydrobromide (MTSEA) (Toronto Research Chemicals, Toronto, Ontario, Canada) (1 mM) was made in ND96 solution immediately before use and bath perfused as well as being co-applied with ATP (1 μM an EC_50_ concentration) via the U-tube.

**The effects of MTS compounds to cysteine mutants**

Cysteine substitution mutants have the added advantage that they can be

probed with methanethiosulfonate (MTS) reagents and this approach has been used widely in structure function studies (Jiang *et al*. 2001; Ennion *et al*. 2002; Rassendren *et al*. 1997; Roberts *et al*. 2007). MTSEA (2-Aminoethyl methanethiosulfonate hydrobromide) can cross the cell membrane freely in its uncharged form. At WT P2X_1_ receptors MTSEA application (1 mM) produced a small potentiation of the response to an EC_50_ concentration of ATP (30.4±4.2%, *n*=3) as reported previously (**Roberts *et al*. 2007**). A similar small effect was observed for D17C, R20C, V22C, L23C, R25C and K28C mutants. MTSEA treatment resulted in significantly larger potentiation for the mutants, M21C, V24C, N26C, K27C and V29C. However for the mutants M21C, N26C, K27C and V29C this potentiation was reversed back to control values following 5 minutes washout of MTSEA suggesting that this effect does not result from the irreversible covalent modification of the introduced cysteine. However for V24C the potentiation was sustained following 5 minutes of washout of the MTSEA.

The mutants Y16C, P19C and G30C were inhibited by MTSEA treatment, and these effects were not reversed following 5 mins washout of MTSEA (Supplementary figure 1). This inhibition showed more time dependence than the effects of MTSEA at WT or other mutants with at least 10 minutes application of MTSEA required before inhibitory effects were seen. The lack of reversal indicates that this results from covalent modification of the introduced cysteine by MTSEA. A large inhibition was observed in the mutant G30C (-45.4±4.9%, *n*=5). Moderate inhibitions were observed for mutants Y16C (-19.1±3.4%, *n*=4) and P19C (-29.9 ±10.6%, *n*=4), the corresponding residues which are associated with the PKC motif and may also be involved in channel regulation. This level of MTSEA inhibition at 20 minutes was the same whether mutants were exposed to ATP or not indicating that the channel does not need to be activated for the introduced cysteines to be modified by MTSEA. These results are similar to those reported for P2X_2_ receptors (Jiang *et al*. 2001).

In addition to the peak amplitudes, 1mM MTSEA had an effect on the time-course of some mutants. For P19C and V29C responses evoked by ATP were almost twice as fast as under control conditions (time for 100 to 50% decay of the current to an EC_50_ concentration of ATP of 46.7 ± 2.4% and 45.7 ± 2.9% of control respectively , *n*=12,18).

The proline at position 19 did not appear to be involved in PMA or glutamate regulation, however, currents at the P2X_1_ P19C mutant receptor were reduced following application of cysteine reactive MTSEA. This is analogous to the effects of methyl methanethiosulfonate at the equivalent mutation at the P2X_2_ receptor (P19C) that reduced ATP evoked currents by ~80%. The effect did not result from a decrease in ATP potency at the P19C P2X_2_ mutant (that would have been expected had there been an effect on ATP binding or channel gating) and it was suggested that this residue may contribute to ionic permeation, although further studies would be needed to substantiate this (Jiang *et al*. 2001). The current mutagenesis studies have shown the contribution of the adjacent tyrosine, aspartate, threonine and arginine residues to regulation and one possibility is that localised changes mediated by these residues in response to PMA could influence ionic permeation through an effect on Pro^19^ and increase P2X_1_ receptor currents by regulating the conductance or the number of available channels.

**Supplementary figure 1. The effects of MTSEA on N-termini cysteine mutants**

**(A).** Representative traces of ATP (1 μM, application period indicated by bar) evoked currents from single *oocytes* of WT and mutant G30C and M21C of P2X_1_ receptors before (open circle) and after the application of 1mM MTSEA (5 minutes application grey circle 20 minutes black circle). The inhibition of G30C by MTSEA was time dependent; in contrast the potentiation of ATP currents at M21C was recorded within 5 minutes of MTSEA application. **(B).** Summary of the maximum percentage changes of the wild type and cysteine mutants by MTSEA. * p<0.05, ** p<0.01, *** p<0.001.  **(C).** Summary of time-dependent effects of MTSEA on WT and cysteine mutant P2X_1_ receptors.

**REFERENCES**

Ennion S. J. and Evans R. J. (2002) P2X(1) receptor subunit contribution to gating revealed by a dominant negative PKC mutant. *Biochem Biophys Res Commun* **291,** 611-616.

Jiang L. H., Rassendren F., Spelta V., Surprenant A. and North R. A. (2001) Amino acid residues involved in gating identified in the first membrane-spanning domain of the rat P2X(2) receptor. *J Biol Chem* **276,** 14902-14908.

Rassendren F., Buell G., Newbolt A., North R. A. and Surprenant A. (1997) Identification of amino acid residues contributing to the pore of a P2X receptor. *EMBO J* **16,** 3446-3454.

Roberts J. A. and Evans R. J. (2007) Cysteine substitution mutants give structural insight and identify ATP binding and activation sites at P2X receptors. *J Neurosci* **27,** 4072-4082.
